# A comparison of medication adherence and viral suppression in antiretroviral treatment-naïve patients with HIV/AIDS depending on the drug formulary

**DOI:** 10.1371/journal.pone.0245185

**Published:** 2021-01-08

**Authors:** Kyung Sun Oh, Euna Han

**Affiliations:** 1 College of Pharmacy, Yonsei Institute of Pharmaceutical Sciences, Yonsei University, Incheon, Republic of Korea; 2 Department of Pharmacy, Inha University Hospital, Incheon, Republic of Korea; Ohio State University, UNITED STATES

## Abstract

Antiretroviral treatment (ART) adherence is highlighted in management of patients living with human immunodeficiency virus. In South Korea, ART medication research has rarely been conducted due to the low economic burden associated with government-funded treatment. This cross-sectional study aimed to compare the pill burden impact between ART regimen compliance and HIV-RNA viral load suppression. Data were collected from 2008 to 2016 at a general hospital in South Korea. A total of 210 HIV/AIDS treatment-naïve patients were grouped as follows: single-tablet regimen (STR, one tablet/day), mild pill burden (two-four tablets/day), and heavy pill burden (≥ five tablets/day). Patients were analyzed according to gender, age at index date, medical insurance type, comorbidities, depression, HIV/AIDS disease burden as indicated by HIV-RNA viral load and CD4, and laboratory variables. In a multivariate logistic regression model, the STR group demonstrated adherence 5.10 times more often than the heavy pill burden group. Females and patients with an initial viral load of 500,000 or more were 0.090- and 0.040-fold less adherent to the ART regimen. Among these patients, 95% or more of the MPR group were 7.38 times more likely to have a lower limit of detection (LLOD) of viral load suppression. The highest initial viral load group was 0.090-fold less likely to have an LLOD than the reference group. These results suggest that a single-tablet regimen could improve medication adherence and the clinical virologic outcome. Therefore, general population research on ART adherence and polypharmacy is needed.

## Introduction

Antiretroviral therapy (ART) is the cornerstone of treatment for patients living with human immunodeficiency virus (HIV) or acquired immune deficiency syndrome (AIDS) [1]. The United Kingdom Collaborative HIV Cohort Study found that HIV-positive patients who achieved viral suppression and had CD4 counts of >350 cells/mm^3^ within one year of antiretroviral treatment (ART) regimen initiation had a normal life expectancy, i.e., it was estimated that a 35-year-old patient would live past the age of 80 years [2]. It is well documented that suboptimal adherence to an ART regimen is associated with a greater risk of developing resistance to antiretroviral agents and increases in morbidity, mortality, and progression to AIDS [3]. The Joint United Nations Program on HIV/AIDS has targeted a goal of 90% medication adherence by 2020 to combat HIV infections [4].

Despite the importance of compliance with an ART regimen, adherence rates are less than optimal, ranging from 60% to 80% [5, 6]. It is recommended to maintain a high compliance rate of 90% or more in managing HIV/AIDS as a chronic disease [1, 7]. Given that all HIV/AIDS patients can be treated through government support in South Korea, they have less economic burden for antiretroviral treatment. This has led to a lack of medication research centered on investigating the relationship between medication adherence and pill burden or viral load suppression.

During the course of our study three Korean-recommended HIV/AIDS guidelines were released—in 2011, 2013, and 2015. These were developed in accordance with the European AIDS Clinical Society and IAS-USA panel strategies [8–10]. The new guidelines included offering antiretroviral treatment to all patients, regardless of their CD4+ T cell counts. Tenofovir disoproxil fumarate (TDF)/emtricitabine (FTC) was introduced as a new and preferred medication in 2013. Elvitegravir/cobicistat/emtricitabine/tenofovir became available only as a single co-formulated tablet, according to the 2015 guideline.

In May 2012, TDF/FTC became available for prescription in HIV/AIDS treatment, and lamivudine (3TC)/abacavir (ABC) became available in August 2012, after its addition to the drug formulary. These simplified ART regimens have made it much easier for patients to take ART medication once a day; this is composed of two tablets of TDF/FTC in addition to efavirenz. Treatment has been further simplified with the addition to the drug formulary of single-tablet regimens such as elvitegravir/cobicistat/emtricitabine/tenofovir (originally listed in April 2014) or dolutegravir/abacavir/lamivudine (listed in March 2016). Therefore, we compared the medication possession ratio (MPR) according to sequential groupings into heavy pill burden (≥ five tablets/day), mild pill burden (two-four tablets/day), and single-tablet regimen (STR, one tablet/day) by chronological drug formulary changes.

We set out to compare ART regimen adherence with pill burden depending on the drug formulary listing of new HIV/AIDS medications. Consequently, the objective of this study was to investigate medication compliance by medication possession ratio (MPR) according to pill burden, as well as by viral load suppression by medication compliance according to MPR.

## Materials and methods

### Data

**Fig 1:**
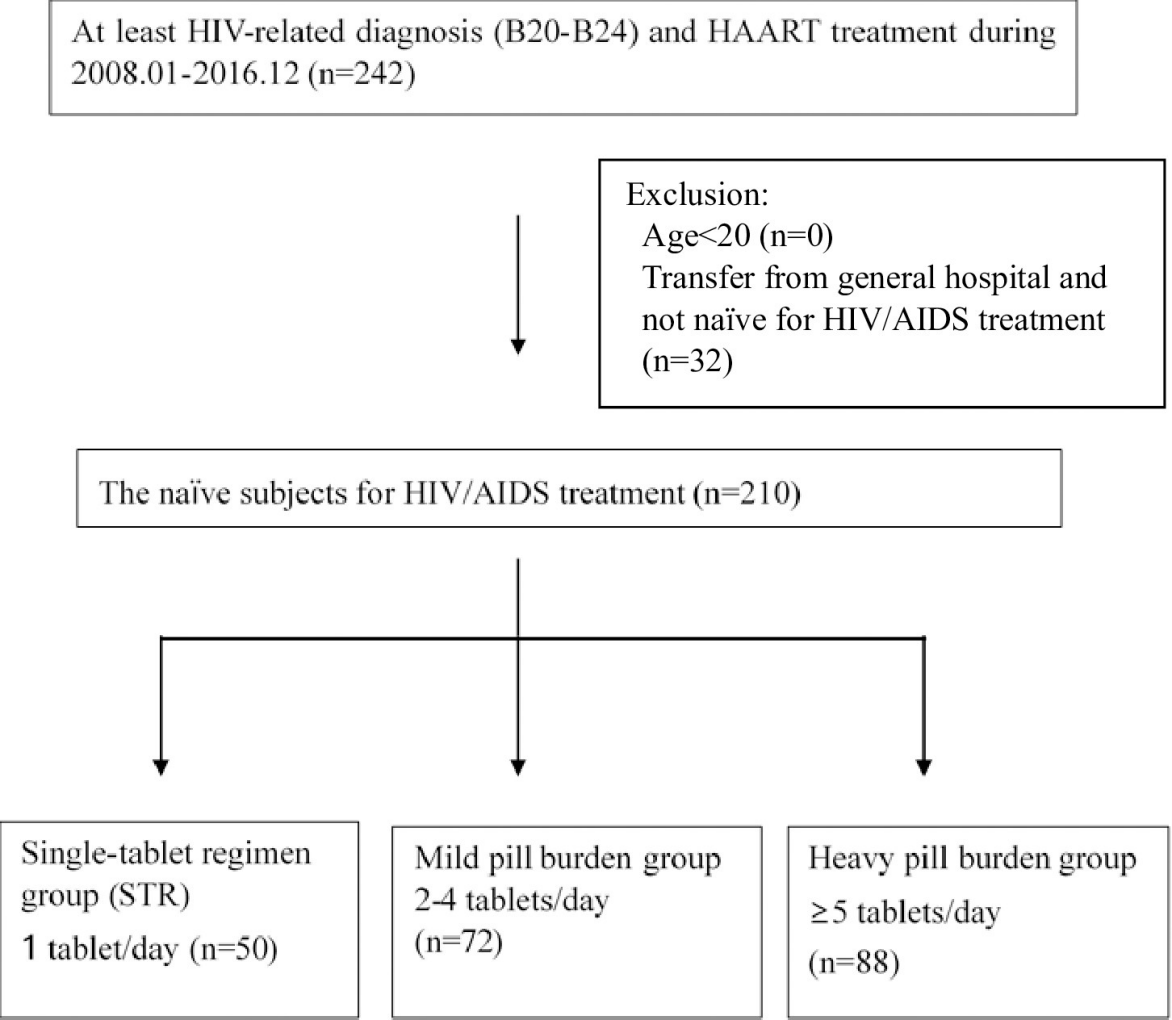
Flow diagram of the study population.

We extracted electronic medical record (EMR) data from a general hospital in South Korea. We selected patients who were at least 20 years of age and had been prescribed an ART regimen between January 01, 2008, and December 31, 2016. A total of 242 patients were initially identified. Of these, the ART-naïve patients were defined based on their transfer from a local public health center or a local clinic and no ART regimen prescription. So, 32 subjects were excluded because their status was not naïve in treatment for HIV/AIDS. The remaining 210 subjects were ART-naïve patients who had been referred from a local clinic to the general hospital for HIV/AIDS treatment. They would be considered for the final analysis. (Fig 1)

### Variables

Patients were categorized into three groups by ART pill count. The magnitude of the pill burden group was measured with the number of tablets according to index date as follows: 1) heavy pill burden group (≥ five tablets/day, the reference group); 2) mild pill burden group (two-four tablets/day), after TDF/ FTC had been added to the drug formulary; and 3) single-tablet regimen group (STR), after the first co-formulated ART, elvitegravir/cobicistat/emtricitabine/tenofovir, had been listed in the drug formulary. The index date was defined as the first date the patient with an HIV/AIDS-related diagnosis (International Classification of Diseases-10 (ICD 10) codes B20-B24) was dispensed a highly active antiretroviral treatment (HAART) regimen. For each group, the number of pills that the patient needed to take per day was counted separately for each antiretroviral treatment. The ART regimens that had been prescribed for a minimum of 30 days after the index date and maintained during the 12–24 week period were assessed. If an ART regimen was changed during the study period, the antiretroviral pill burden was determined to be the average pill number.

We analyzed two outcomes: the degree of ART regimen adherence and viral load suppression. Adherence to the ART regimen was measured with the MPR, which is a proxy for medication compliance by pharmacy refill data. The MPR was calculated as the total number of days from the index date to the date of the viral load result and was used as the denominator. During that period, the number of days each patient had the ART available was used as a numerator [11, 12]. The MPR was calculated as follows.

$$\frac{\sum \;\text{Total}\;\text{number}\;\text{of}\;\text{days}\;\text{available}\;\text{with}\;\text{antiretroviral}\;\text{treatment}}{\sum \;\text{Total}\;\text{number}\;\text{of}\;\text{days}\;\text{between}\;\text{the}\;\text{index}\;\text{date}\;\text{and}\;\text{the}\;\text{date}\;\text{of}\;\text{HIV-RNA}\;\text{result}}$$

Patients with an MPR of 95% or higher were considered adherent to the medication regimen [13–15]. The MPR was cut off at “1” for patients with higher than 100% adherence.

After ART medication was initiated, confirmation of the HIV-RNA viral load between week12 and week24 was recommended [8, 10]. The antiretroviral effect is confirmed by the lower limit of detection (LLOD) result after ART initiation. So, the viral load suppression analysis was conducted on a subset of patients who had HIV-RNA levels both on the pre-index date and during the 12–24-week period. Virologic suppression was defined as the state in which the HIV-RNA polymerase chain reaction (HIV-RNA PCR) level during the 12–24 weeks was not detected after the ART regimen was given to the patients. Virologic suppression was dichotomized with a dummy indicator representing 20 copies/ml or less defined as the lower limit of detection, to determine whether virologic suppression was achieved [16–18].

The Anderson model has been evaluated as suitable for analyzing predictive factors for the use of medical and social services. Therefore, we referenced several covariates to account for patient characteristics (predisposing factors: gender and age at index date; enabling factors: type of medical insurance; vulnerable need: depression, comorbidities) that was frequently cited in past HIV/AIDS articles [19, 20]. The HIV/AIDS disease burden was measured using the pre-index date HIV-RNA viral load and CD4 as well as laboratory variables [5, 14]. In Korea, 97% of the population must register with the mandatory National Health Insurance, and the remaining 3%, those in the lowest socioeconomic level, are covered by National Medical Aid. Given this, we used the medical insurance type to account for their financial status. As a proxy for comorbidities, we collected data on syphilis and viral hepatitis (Hepatitis B and Hepatitis C) for infectious diseases. We included all kinds of neoplasm identified at the referred outpatient visit [21]. A syphilis co-infection was confirmed by ICD-10 codes (A50-A53) and a benzathine penicillin G prescription. Viral hepatitis B or hepatitis C, chronic infectious diseases, were confirmed through ICD-10 codes (B18 or K73), and viral hepatitis medication treatment. Malignancy, including leukemia and lymphoma, was defined by diagnosis code (ICD-10 codes C00-C96; see S1 Table) and chemotherapy. The presence of depression was defined by a psychiatric consultation record and the WHO ATC code for an N06A antidepressant or N05B anxiolytics prescription [22]. As baseline characteristics, we used the HIV/AIDS disease burden and clinical laboratory data on creatinine, fasting blood glucose, lipid panel (total cholesterol, low-density lipoprotein (LDL), triglycerides (TG), and high-density lipoprotein (HDL) within the four weeks before the index date. We also created categorical variables to account for the HIV/AIDS disease burden. The CD4 count was divided into three levels: <200 (requiring prophylactic antibiotics), 200–349, and ≥350. Finally, the pre-index viral load was classified into four groups: less than 10 000, 10 000-<100 000, 100 000-<500 000, and more than 500 000.

### Statistical analysis

This was a cross-sectional study. The patients’ demographic and clinical characteristics were compared across the three pill burden groups using the χ2 test, Fisher’s exact test, or analysis of variance, as appropriate. Logistic regression models were performed to identify the factors associated with the MPR and to evaluate the difference in viral load suppression between MPR 95% and less than according to pill burden groups and baseline covariates. All analyses were performed using STATA 15 (Stata Corp, College Station, TX, USA).

### Ethics

This study was approved by the Institutional Review Board of Inha University Hospital (INHAUH 2018-11-011-002). All information that could identify the subject, such as name and registration number, was deleted from the electronic medical records for data confidentiality and the subject’s anonymity. The extracted data was generated and managed by password-protected files, to which only one researcher had access. All data, including statistical analyses and results, were stored in one designated computer set with a security number, which was blocked from the external network. An informed consent waiver was approved by the Institutional Review Board of Inha University Hospital.

## Results

A total of 242 HIV/AIDS patients were prescribed antiretroviral therapy in the years 2008–2016. Of these, 32 subjects were excluded because they were referred from other general hospitals with a non-naïve HIV/AIDS status. The remaining 210 subjects were ART-naïve patients and were included in the final analysis. The baseline characteristics of these 210 subjects are presented in Table 1.

**Table 1:** Baseline characteristics of subjects (n = 210, n, (%) or mean ±SD).

Variables	STR^1)^ (n = 50, n (%))	Mild pill group ^1)^ (n = 72, n (%))	Heavy pill group^1)^ (n = 88, n (%))	p-value
Main independent variable				
Antiretroviral treatment pill burden	1	2.9 ± 0.8	7.1 ± 2.0	<0.001
Dependent variables				
Medication possession ratio				
≥ 95%	47 (94.0)	55 (76.4)	62 (70.4)	0.002
< 95%	3 (6.0)	17 (23.6)	26 (29.6)	
Viral load suppression (LLOD, lower limit of detection)				
Yes	39 (78.0)	47 (65.3)	45 (37.5)	0.011
No	8 (16.0)	20 (27.8)	33 (42.3)	
Covariates				
Pre-index viral load				
<10,000	8(16.0)	7 (9.7)	7 (8.1)	0.200
10,000 - <100,000	29 (58.0)	29 (40.3)	40 (45.9)	
100,000 - <500,000	10 (20.0)	28 (38.9)	30 (34.5)	
≥500,000	3 (6.0)	8 (11.1)	10 (11.5)	
Pre-index CD4 count				
<200	17 (34.0)	32 (44.4)	47 (54.7)	0.017
200–349	19 (38.0)	24 (33.3)	32 (37.2)	
≥350	14 (28.0)	16 (22.3)	7 (8.1)	
Gender				
Male	48 (96.0)	69 (95.8)	82 (93.2)	0.729
Female	2 (4.0)	3 (4.2)	6 (6.8)	
Age at index date	37.2 ± 11.0	43.6 ± 12.6	43.5 ± 13.1	0.008
Medical insurance type				
National Health Insurance	48 (96.0)	59 (81.9)	73 (82.9)	0.043
National Medical Aid	2 (4.0)	13 (18.1)	15 (17.1)	
Depression	9 (18.0)	13 (18.1)	15 (17.1)	0.983
Comorbidity				
Syphilis	7 (14.0)	8 (11.1)	18 (20.5)	0.252
Viral hepatitis	1 (2.0)	2 (2.8)	3 (3.4)	0.891
Malignancy	0 (0.0)	3 (4.2)	3 (3.4)	0.400
Clinical Laboratory				
Creatinine	0.93 ± 0.15	1.01 ± 0.34	0.98 ± 0.17	0.171
Fasting Blood Glucose	105.9 ± 52.2	107.2 ± 32.8	106.9 ± 31.3	0.982
Total Cholesterol	147.7 ± 32.8	148.7 ± 35.4	156.6 ± 32.4	0.212
Triglycerides	136.9 ± 98.9	165.4 ± 95.5	174.1 ± 106.6	0.129
Low-density lipoprotein	92.0 ± 27.8	89.6 ± 27.2	94.1 ± 25.7	0.633
High-density lipoprotein	39.6 ± 14.1	36.2 ± 10.8	40.1 ± 12.6	0.159

1) STR: Single tablet regimen group, Mild pill group: two-four tablets/day, Heavy pill group: ≥ five tablets/day.

P-value estimated using χ2 test, Fisher’s exact test in categorical variables and analysis of variance in continuous variables.

**Fig 2:**
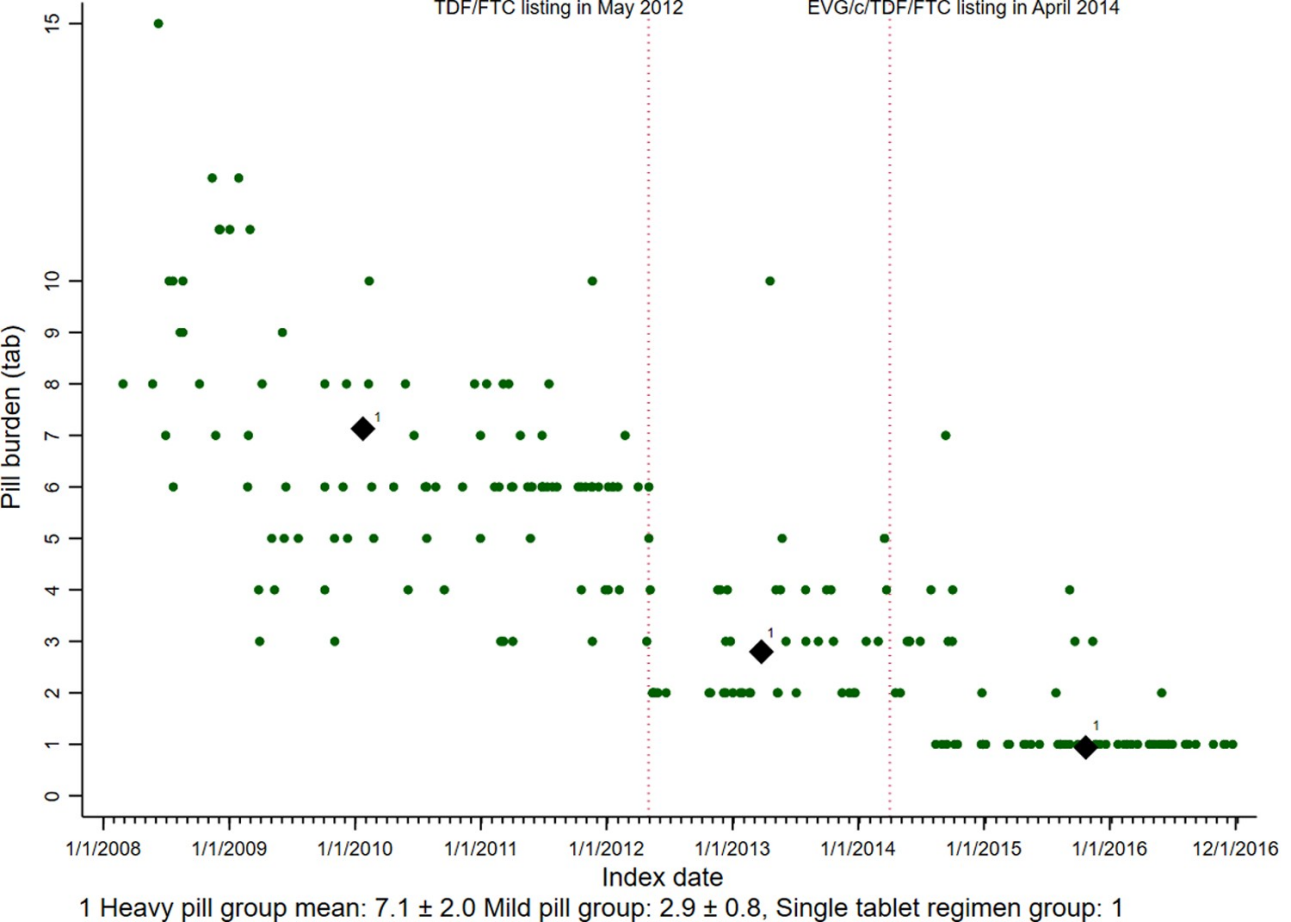
Antiretroviral pill burden changes over time.

Males predominated in all three different pill burden groups at an average of 93.2% to 96%. No statistically significant differences were found among the three groups for pre-index viral load, presence of depression, comorbidities, and all clinical laboratory data (total cholesterol, triglycerides (TG), LDL, and HDL). In the case of pre-index viral load, there was one patient without a pre-index HIV-RNA PCR, but their HIV/AIDS positive result was indicated only by the ELISA method (reference negative) [23]. In addition, there is no statistically significant difference at pre-index viral load; it showed a trend with much lower pre-index viral load over time. The average age at the beginning of the ART regimen was 43.5±13.1 for the heavy pill burden group, 43.6 ±12.6 for the mild pill burden group, and 37.2 ±11.0 for the single-tablet regimen group (p = 0.008)). A posthoc test showed that the STR group was statistically different from the heavy pill burden group and the mild pill burden group. The proportion of National Medical Aid in the STR group was less than that of the heavy pill burden group because of their younger age as of the index date. (p = 0.043) These differences in the actual pill burden appeared among the three groups. The mean of the ART pill numbers per day was 7.1 ± 2.0, 2.9 ± 0.8, and one tablet for the heavy pill burden group, mild pill burden group, and the single-tablet regimen group. (p <0.001), respectively. The ART actual pill changes over time are plotted in Fig 2. When a new blockbuster HIV/AIDS medication was added to the drug formulary, the recommended pill burden for patients gradually decreased. The number of patients that had ART regimen changes within wk12-24 was 27. Twenty-three patients had records indicating the reason for the treatment change, and four were without valid reasons for it. The rate of patients who shifted to a different formulation owing to adverse effects and lab abnormality is 11.3% for the heavy-pill group, 7.0% for the mild-pill group, and 6.0% for the single-tablet group. This is 8.6% of all 210 persons in the sample (see S2 Table).

Among the three groups, the heavy pill burden group of 47 patients (54.7%) had a CD4 count below the 200 required for prophylactic antibiotic treatment, whereas only seven (8.1%) patients had a CD4 count >350. However, in the single-tablet regimen group, the proportion of CD4 count below the 200 was 34%, and the CD4 count over the 350 was 28%, relatively (p = 0.017). These results may reflect guideline changes, including the offer of antiretroviral treatment to all patients regardless of their CD4+ T cell counts in the 2013 guidelines. Of the three groups, the total of syphilis-infected patients was 33 patients; the heavy pill group had 18 patients (20.5%), eight patients (11.1%) were in the mild pill burden group, and seven patients (14%) were in STR, respectively. The proportion of patients who were prescribed antidepressants or anti-anxiety medications for depression was similar, between 17% and 18% among the three groups, regardless of duration.

Univariate and multivariate logistic analysis indicated that gender, pre-index viral load, and antiretroviral treatment pill burden were associated with the MPR, as shown in Table 2. The univariate analysis showed that females were less likely to be adherent (unadjusted OR = 0.21, 95% CI, 0.01–0.72) than males. Patients with a higher initial viral load were less likely to be adherent (unadjusted OR = 0.08, 95% CI, 0.01–0.69). The single-tablet regimen was 6.57 times more likely to be adhered to than that of the heavy pill burden group (unadjusted OR = 6.57, 95% CI, 1.88–23.0). The multivariate logistic analysis also showed patterns similar to the findings of the univariate analyses: the single-tablet regimen was 5.10 times more likely to be adhered to than that of the heavy pill burden group (adjusted OR = 5.10, 95% CI, 1.32–19.7). Female patients were 0.09-fold less likely to adhere to the ART regimen (95% CI, 0.02–0.44) as compared with male patients. Those with an initial viral load of 500,000 or more also were 0.040-fold less likely to adhere to (95% CI, 0.01–0.56) than the reference group with an initial viral load of less than 10,000 (Table 2).

**Table 2:** Factors associated with MPR (medication possession ratio) (n = 210).

Variables	Univariate logistic analysis OR (95% CI) MPR ≥ 95% (n = 164)	Multivariate logistic analysis OR (95% CI) MPR ≥ 95% (n = 164)
Antiretroviral treatment		
Pill burden		
Heavy pill burden group^1)^	Reference	Reference
Mild pill burden group^1)^	1.36 (0.67–2.76)	1.24 (0.56–2.75)
Single tablet regimen^1)^	6.57 (1.88–23.0)	5.10 (1.32–19.7)
Gender		
Male	Reference	Reference
Female	0.21 (0.01–0.72)	0.09 (0.02–0.44)
Age at index date	0.99 (0.97–1.02)	0.99 (0.96–1.02)
Medical insurance type		
National Health Insurance	Reference	Reference
National Medical Aid	0.50 (0.21–1.16)	0.81 (0.29–2.26)
Pre-index viral load		
<10,000	Reference	Reference
10,000 - <100,000	0.18 (0.02–1.46)	0.10 (0.01–1.17)
100,000 - <500,000	0.15 (0.01–1.24)	0.09 (0.01–1.11)
≥500,000	0.08 (0.01–0.69)	0.04 (0.01–0.56)
Pre-index CD4 count		
<200	Reference	Reference
200–349	1.59 (0.75–3.38)	0.91 (0.39–2.14)
≥350	1.21 (0.49–2.99)	0.89 (0.26–2.81)
Depression	0.71 (0.31–1.60)	0.59 (0.24–1.48)
Comorbidity		
Syphilis	0.85 (0.34–2.05)	0.83 (0.29–2.33)
Viral hepatitis	0.27 (0.05–1.37)	0.12 (0.01–0.89)
Malignancy	0.27 (0.05–1.37)	0.49 (0.07–3.62)

1) STR: Single tablet regimen group, Mild pill group: two-four tablets/day, Heavy pill group: ≥ five tablets/day.

A binary logistic regression analysis on viral load suppression revealed that the pre-index viral load and medication compliance of 95% or higher were important factors, as shown in Table 3. We obtained HIV-RNA PCR results for 192 patients at both the pre-index date and during 12–24 weeks. In the 12–24-week test, 118 patients with a 95% or higher MPR had an LLOD, and 33 patients failed, whereas in the less than the 95% MPR group, only 13 patients achieved LLOD; 28 patients did not.

**Table 3:** Factors associated with viral load suppression (Lower Limit of Detection (LLOD)) (n = 192).

	Univariate logistic analysis viral load suppression (LLOD), (95% CI),	Multivariate logistic analysis viral load suppression (LLOD), OR (95% CI)
Variables	Yes (n = 131)	Yes (n = 131)
Medication possession ratio		
<95%	Reference	Reference
≥95%	7.74 (3.59–16.5)	7.38 (2.84–19.2)
Antiretroviral treatment		
Pill burden		
Heavy pill burden group^1)^	Reference	Reference
Mild pill burden group^1)^	1.72 (0.86–3.43)	1.80 (0.75–4.28)
STR^1)^	3.56 (1.48–8.65)	1.78 (0.60–5.27)
Gender		
Male	Reference	Reference
Female	0.36 (0.11–1.24)	0.34 (0.07–1.59)
Age at index date	0.99 (0.97–1.01)	0.99 (0.96–1.02)
Medical insurance type		
National Health Insurance	Reference	Reference
National Medical Aid	0.92 (0.40–2.10)	0.99 (0.32–3.09)
Pre-index viral load		
<10,000	Reference	Reference
10,000 - <100,000	0.40 (0.09–1.90)	0.86 (0.18–4.16)
100,000 - <500,000	0.11 (0.02–0.49)	0.22 (0.04–1.09)
≥500,000	0.06 (0.01–0.36)	0.09 (0.01–0.59)
Initial CD4 count		
<200	Reference	Reference
200–349	1.32 (0.68–2.61)	0.76 (0.32–1.82)
>350	2.54 (1.00–6.45)	1.66 (0.47–5.87)
Depression	0.92 (0.41–2.03)	1.07 (0.37–3.04)
Comorbidity		
Syphilis	1.82 (0.74–4.47)	2.18 (0.64–7.44)
Viral hepatitis	0.46 (0.06–3.33)	0.96 (0.10–9.67)
Malignancy	0.93 (0.17–5.21)	4.05 (0.25–65.1)

1) STR: Single tablet regimen group, Mild pill group: two-four tablets/day, Heavy pill group: ≥ five tablets/day.

In the unadjusted analysis, a group with 95% or higher MPR was 7.74 times more likely to have viral load suppression (95% CI, 3.59–16.5). In contrast, a high pre-index viral load (≥500,000) was 0.060-fold less likely to have virologic suppression (95% CI, 0.01–0.36). Such results were parallel with the multivariate logistic analysis. Patients with a 95% or higher MPR were 7.38 times more likely to have viral load suppression than those with an MPR of lower than 95% (95% CI, 2.84–19.2). The group with a pre-index viral load of 500,000 or more was 0.09-fold less likely to have viral load suppression (95% CI, 0.01–0.59) than that with a pre-index viral load of less than 10,000. In contrast, unlike the results of the univariate logistic regression analysis, the antiretroviral treatment pill burden group did not show a statistically significant association with the viral load suppression in multivariate logistic regression analysis (Table 3).

## Discussion

We investigated the relation between pill burden and medication adherence and viral load suppression. In particular, we assessed whether a gradual change in pill burden, based on drug formulary changes from more than five tablets a day (heavy pill burden) to two-four tablets (mild pill burden) or a single-tablet level, was associated with a higher rate of medication adherence and virologic suppression.

Our findings showed that a lower pill burden, a lower HIV-RNA level, and male patients were associated with higher medication adherence. Patients who had a higher initial viral level had a lower MPR. In addition, the MPR of the lower pill burden group as STR was higher than that of the heavy pill burden group. These are parallel with the results of other global studies comparing the MPR of STR and MTR for drug compliance [7, 24, 25]. In this study, female patients were associated with lower medication adherence. This also coincides with the results of previous studies on the Korean population in which the female group characterized by lower medication adherence [5, 26]. In addition, this study found that good medication adherence (95% or higher MPR) and lower initial viral load indicated a higher rate of viral load suppression. Of the total 210 patients, the 118 patients in the 95% or higher MPR group achieved viral load suppression. In the lower than 95% MPR group, only 13 patients reached an LLOD. According to previous studies, the MPR is closely associated with the virologic failure rate [27–30]. During the study period from 2008 to 2016, the rate of HIV/AIDS patients with depression did not change significantly, remaining between 17% to 18%. According to previous studies about depression and AIDS, social stigma related to HIV/AIDS causes depression and anxiety, which may not only provoke suicidal ideation but reduce medication compliance [31, 32]. To reduce the number of HIV/AIDS patients with depression and improve ART compliance, HIV/AIDS-related knowledge should be increased, and HIV/AIDS-related stigma must be reduced.

This study has several limitations. First, we analyzed a small sample of 210 individuals, which is too small to represent the general Korean population. From 2008 to 2016, an average number of 20–30 HIV/AIDS-naïve patients were enrolled in the institution from which our data were extracted, excluding patients who had transferred while already in HIV/AIDS treatment. Considering that 1,191 new HIV/AIDS subjects were registered in 2017 across Korea, this means that our study sample accounted for only approximately 2.5% of all newly-enrolled HIV/AIDS patients nationwide [33]. Furthermore, 18 patients or approximately 8.5% of the 210 subjects who were initially included in our study did not have outpatient visits during weeks 12–24; therefore, they were excluded from the final virologic suppression analysis owing to a lack of HIV-RNA PCR data during that period. However, as the majority of the 18 patients were successfully treated, showing a lower viral load of fewer than 100 copies/ml before week 12 (two patients obtained LLOD earlier within wk4 and five patients’ HIV-RNA level was 20–99 copies/ml), so their outpatient follow-up visit interval was extended to more than three months. As for the 18 patients, there were 13 patients with a HIV-RNA PCR before wk12, four patients with LLOD results after wk24, and only one patient without a pre-index HIV-RNA viral load. Additionally, five of the total 18 patients obtained LLOD at the first HIV-RNA PCR after taking ART, with two obtaining LLOD earlier within wk4. However, this does not make any significant statistical difference in MPR and viral load suppression if we conduct additional analysis of the total of 210 subjects. This study used the MPR as the indicator of medication adherence because it is appropriate for HIV/AIDS patients who generally receive multiple ART medications in the same outpatient visit. However, the MPR has its own limitation in that it can be measured only based on the ART dispensing record. Thus, it is not possible to ascertain whether patients actually take their ART medication [29, 34]. Finally, second-generation antiretroviral medications or single-tablet regimens have lower toxicities and lower viral resistance rates, which contribute to better medication adherence and the viral suppression effect. The improved effectiveness of newer ART medication is consistent with our research finding that compared to the heavy pill burden group, the single-tablet group sustained a higher level of medication adherence and viral load suppression.

Despite these limitations, our study provides a significant finding regarding the relationship between pill burden and ART adherence. According to the results, the lower pill burden represented by taking STR and the lower HIV/AIDS disease burden represented by the lower initial viral load were associated with better medication adherence and virologic suppression. These results suggest that the STR could improve medication adherence and clinical virologic outcomes, as we expected. As HIV/AIDS patients continue to live longer, it is becoming increasingly important to manage medication adherence and concomitant medications effectively; therefore, further research on ART adherence and polypharmacy is needed for the general population.

## Supporting information

S1 Table ICD-10 codes associated with covariates. (DOCX)

S2 Table The number of patients who had an antiretroviral treatment change within wk12-24. (DOCX)

## References

[1] Günthard HF, Saag MS, Benson CA, et al Antiretroviral drugs for treatment and prevention of hiv infection in adults: 2016 recommendations of the international antiviral society–usa panel. JAMA. 2016;316(2):191–210. 10.1001/jama.2016.8900 27404187 PMC5012643

[2] May M, Gompels M, Sabin C. Life expectancy of HIV-1-positive individuals approaches normal conditional on response to antiretroviral therapy: UK Collaborative HIV Cohort Study. Journal of the International AIDS Society. 2012;15(Suppl 4):18078.

[3] Cheng Y, Sauer B, Zhang Y, Nickman NA, Jamjian C, Stevens V, et al Adherence and virologic outcomes among treatment-naive veteran patients with human immunodeficiency virus type 1 infection. Medicine (Baltimore). 2018;97(2):e9430 10.1097/MD.0000000000009430 29480831 PMC5943894

[4] HIV/AIDS JUNPo, HIV/Aids JUNPo. 90-90-90: an ambitious treatment target to help end the AIDS epidemic. Geneva: Unaids 2014.

[5] Kim J, Lee E, Park BJ, Bang JH, Lee JY. Adherence to antiretroviral therapy and factors affecting low medication adherence among incident HIV-infected individuals during 2009–2016: A nationwide study. Scientific Reports. 2018;8(1):3133 10.1038/s41598-018-21081-x 29453393 PMC5816616

[6] Cohen CJ, Meyers JL, Davis KL. Association between daily antiretroviral pill burden and treatment adherence, hospitalisation risk, and other healthcare utilisation and costs in a US medicaid population with HIV. BMJ Open. 2013;3(8). 10.1136/bmjopen-2013-003028 PMC3733306 23906955

[7] Cheng Y, Nickman NA, Jamjian C, Stevens V, Zhang Y, Sauer B, et al Predicting poor adherence to antiretroviral therapy among treatment-naive veterans infected with human immunodeficiency virus. Medicine (Baltimore). 2018;97(2):e9495 10.1097/MD.0000000000009495 29480838 PMC5943852

[8] Clinical Guidelines for the Diagnosis and Treatment of HIV/AIDS in HIV-infected Koreans. Infection and Chemotherapy. 2011;43(2).

[9] Korean Society for A. The 2013 Clinical Guidelines for the Diagnosis and Treatment of HIV/AIDS in HIV-Infected Koreans. Infect Chemother. 2013;45(4):455–61. 10.3947/ic.2013.45.4.455 24475362 PMC3902823

[10] Korean Society for A. The 2015 Clinical Guidelines for the Diagnosis and Treatment of HIV/AIDS in HIV-Infected Koreans. Infect Chemother. 2015;47(3):205–11. 10.3947/ic.2015.47.3.205 26483998 PMC4607777

[11] Fairman KA, Motheral BJJomcp. Evaluating medication adherence: which measure is right for your program? 2000;6(6):499–506.

[12] Steiner JF, Prochazka AV. The assessment of refill compliance using pharmacy records: methods, validity, and applications. Journal of Clinical Epidemiology. 1997;50(1):105–16. 10.1016/s0895-4356(96)00268-5 9048695

[13] Paterson DL, Swindells S, Mohr J, Brester M, Vergis EN, Squier C, et al Adherence to protease inhibitor therapy and outcomes in patients with HIV infection. Annals of Internal Medicine. 2000;133(1):21–30. 10.7326/0003-4819-133-1-200007040-00004 10877736

[14] Sutton SS, Magagnoli J, Hardin JW. Odds of viral suppression by single‐tablet regimens, multiple‐tablet regimens, and adherence level in HIV/AIDS patients receiving antiretroviral therapy. Pharmacotherapy: The Journal of Human Pharmacology and Drug Therapy. 2017;37(2):204–13. 10.1002/phar.1889 28028855

[15] Tandon N, Mao J, Shprecher A, Anderson AJ, Cao F, Jiao X, et al Compliance with clinical guidelines and adherence to antiretroviral therapy among patients living with HIV. Curr Med Res Opin. 2019;35(1):63–71. 10.1080/03007995.2018.1519499 30173561

[16] DHHS guideline_Guidelines for the use of antiretroviral agents in HIV-1-infected adults and adolescents.pdf.

[17] Gunthard HF, Saag MS, Benson CA, del Rio C, Eron JJ, Gallant JE, et al Antiretroviral Drugs for Treatment and Prevention of HIV Infection in Adults: 2016 Recommendations of the International Antiviral Society-USA Panel. JAMA. 2016;316(2):191–210. 10.1001/jama.2016.8900 27404187 PMC5012643

[18] Thompson MA, Aberg JA, Hoy JF, Telenti A, Benson C, Cahn P, et al Antiretroviral treatment of adult HIV infection: 2012 recommendations of the International Antiviral Society–USA panel. JAMA. 2012;308(4):387–402. 10.1001/jama.2012.7961 22820792

[19] Anderson JG. Health services utilization: framework and review. Health Serv Res. 1973;8(3):184–99. 4593850 PMC1071757

[20] Anthony MN, Gardner L, Marks G, Anderson-Mahoney P, Metsch LR, Valverde EE, et al Factors associated with use of HIV primary care among persons recently diagnosed with HIV: examination of variables from the behavioural model of health-care utilization. AIDS Care. 2007;19(2):195–202. 10.1080/09540120600966182 17364398

[21] Lee E, Kim J, Bang JH, Lee JY, Cho S-i. Association of HIV-syphilis coinfection with optimal antiretroviral adherence: a nation-wide claims study. AIDS Care. 2020;32(5):651–5. 10.1080/09540121.2019.1686602 31690082

[22] Yun LWH, Maravi M, Kobayashi JS, Barton PL, Davidson AJ. Antidepressant treatment improves adherence to antiretroviral therapy among depressed HIV-infected patients. JAIDS Journal of Acquired Immune Deficiency Syndromes. 2005;38(4):432–8. 10.1097/01.qai.0000147524.19122.fd 15764960

[23] Kee M-K, Lee J-H, Whang J, Kim SS. Ten-year trends in HIV prevalence among visitors to public health centers under the National HIV Surveillance System in Korea, 2000 to 2009. BMC Public Health. 2012;12(1):831 10.1186/1471-2458-12-831 23020818 PMC3509016

[24] Zhou S, Martin K, Corbett A, Napravnik S, Eron J, Zhu Y, et al Total daily pill burden in HIV-infected patients in the southern United States. AIDS Patient Care STDS. 2014;28(6):311–7. 10.1089/apc.2014.0010 24901464 PMC4180528

[25] Guaraldi G, Menozzi M, Zona S, Calcagno A, Silva AR, Santoro A, et al Impact of polypharmacy on antiretroviral prescription in people living with HIV. J Antimicrob Chemother. 2017;72(2):511–4. 10.1093/jac/dkw437 27834193

[26] Yang HJ, Bang JH. Factors associated with medication adherence in patients with human immunodeficiency virus in South Korea. AIDS Care. 2017;29(10):1315–9. 10.1080/09540121.2017.1282104 28127987

[27] Scott Sutton S, Magagnoli J, Hardin JW. Impact of Pill Burden on Adherence, Risk of Hospitalization, and Viral Suppression in Patients with HIV Infection and AIDS Receiving Antiretroviral Therapy. Pharmacotherapy. 2016;36(4):385–401. 10.1002/phar.1728 26923931

[28] Gordon LL, Gharibian D, Chong K, Chun H. Comparison of HIV Virologic Failure Rates Between Patients with Variable Adherence to Three Antiretroviral Regimen Types. AIDS Patient Care STDS. 2015;29(7):384–8. 10.1089/apc.2014.0165 26114665

[29] Cooke CE, Lee HY, Xing S. Adherence to antiretroviral therapy in managed care members in the United States: a retrospective claims analysis. J Manag Care Pharm. 2014;20(1):86–92. 10.18553/jmcp.2014.20.1.86 24372462 PMC10437878

[30] Yager J, Faragon J, McGuey L, Hoye-Simek A, Hecox Z, Sullivan S, et al Relationship Between Single Tablet Antiretroviral Regimen and Adherence to Antiretroviral and Non-Antiretroviral Medications Among Veterans’ Affairs Patients with Human Immunodeficiency Virus. AIDS Patient Care STDS. 2017;31(9):370–6. 10.1089/apc.2017.0081 28771023

[31] Kang CR, Bang JH, Cho S-I, Kim KN, Lee H-j, Ryu BY, et al Suicidal ideation and suicide attempts among human immunodeficiency virus-infected adults: differences in risk factors and their implications. AIDS Care. 2016;28(3):306–13. 10.1080/09540121.2015.1093593 26444525

[32] Akincigil A, Wilson IB, Walkup JT, Siegel MJ, Huang C, Crystal S. Antidepressant treatment and adherence to antiretroviral medications among privately insured persons with HIV/AIDS. AIDS and Behavior. 2011;15(8):1819–28. 10.1007/s10461-011-9938-6 21484284 PMC3719048

[33] Choi BY, Choi JY, Han SH, Kim SI, Kee M-K, Kim MJ, et al Korea HIV/AIDS Cohort Study: study design and baseline characteristics. Epidemiology and Health. 2018;40:e2018023-e. 10.4178/epih.e2018023 30134649 PMC6178365

[34] Osterberg L, Blaschke T. Adherence to Medication. New England Journal of Medicine. 2005;353(5):487–97. 10.1056/NEJMra050100 16079372

