# Supplementary material for: A comparison of medication adherence and viral suppression in antiretroviral treatment-naïve patients with HIV/AIDS depending on the drug formulary
Source: PLoS One. 2021 Jan 8;16(1):e0245185. doi: 10.1371/journal.pone.0245185 (PMC7793268; doi:10.1371/journal.pone.0245185)
Supplement: S1 Table — (DOCX) [file pone.0245185.s001.docx]

**S1 Table. ICD-10 codes associated with covariates.**

| Variables | ICD-10 codes |
| --- | --- |
| Syphilis | A50-A53 |
| Viral hepatitis | B18, K73 |
| Any malignancy, including leukemia and lymphoma | C00-C26, C30 -C34, C37-C41, C43, C45-C58, C60-C76., C81-C85, C88, C90-C96 |
| Metastatic solid tumor | C77-C80 |
