# Supplementary material for: A comparison of medication adherence and viral suppression in antiretroviral treatment-naïve patients with HIV/AIDS depending on the drug formulary
Source: PLoS One. 2021 Jan 8;16(1):e0245185. doi: 10.1371/journal.pone.0245185 (PMC7793268; doi:10.1371/journal.pone.0245185)
Supplement: S2 Table — (DOCX) [file pone.0245185.s002.docx]

**S2 Table. The number of patients who had an antiretroviral treatment change within wk12-24.**

| Reason for change in ART | Heavy-pill group^1)^ | Mild-pill group^1)^ | Single-tablet regimen group^1)^ |
| --- | --- | --- | --- |
| Adverse effects  (rash, diarrhea) | 8 | 3 | 3 |
| Lab abnormality | 2 | 2 | 0 |
| Pill compliance | 3 | 0 | 0 |
| Out of stock | 1 | 0 | 0 |
| Storage (refrigeration) | 1 | 0 | 0 |
| Total number of ART changes | 15 | 5 | 3 |
| Total sample | 88 | 72 | 50 |
| ART change potentially due to adverse effects (n, % of the total sample) | 10 (11.3%) | 5 (7.0%) | 3 (6.0%) |

1) Single-tablet regimen group: one tablet/day, Mild-pill group: two-four tablets/day, Heavy-pill group: ≥ five tablets/day
